# Sports Emotional Intelligence and Exercise Adherence in Adolescents: The Chain Mediating Role of Exercise Enjoyment and Exercise Identity

**DOI:** 10.3390/jintelligence14090222

**Published:** 2026-09-15

**Authors:** Donghuan Bai, Xuerui Li, Pengwei Song, Jia Zhang

**Affiliations:** 1 School of Physical Education, Huaibei Normal University, Huaibei 235000, China; 2 School of Physical Education, Chongqing University, Chongqing 400044, China; 3 School of Physical Education, Guangxi Science & Technology Normal University, Laibin 546199, China

**Keywords:** sports emotional intelligence, exercise enjoyment, exercise identity, exercise adherence, adolescent

## Abstract

**Objective**: This study explores the relationship between sports emotional intelligence and exercise adherence in adolescents and constructs a sequential mediation model through the mediating roles of exercise enjoyment and exercise identity. **Methods**: Using a school-based cross-sectional survey, 1278 adolescents aged 12–18 years from Anhui, Henan, Guangxi, and Chongqing were recruited and completed the Sports Emotional Intelligence Scale, Exercise Enjoyment Scale, Exercise Identity Scale, and Exercise Adherence Scale. Data were analyzed using Mplus 8.1 and SPSS 25.0. Common method bias was assessed with Harman’s single-factor test. The measurement model was tested via confirmatory factor analysis, and chain indirect effects were examined using the bootstrap method. **Results**: The measurement model demonstrated good fit, and all scales showed satisfactory reliability and validity. (1) Sports emotional intelligence was significantly associated with exercise adherence (β = 0.44, *p* < 0.001); (2) exercise enjoyment (indirect effect = 0.109) and exercise identity (indirect effect = 0.094) each mediated the relationship, and the sequential path from sports emotional intelligence through exercise enjoyment to exercise identity and then to adherence was also significant (indirect effect = 0.079), with all bias-corrected bootstrap 95% confidence intervals excluding 0. **Conclusion**: Sports emotional intelligence was positively associated with exercise adherence in adolescents, and this association was partly explained by indirect associations involving exercise enjoyment and exercise identity. The findings suggest that affective valuation and exercise-related self-concept are important correlates within the proposed model.

## 1. Introduction

During adolescence, physical activity becomes less embedded in daily routines and increasingly depends on young people’s own choices. This transition is important because initiating exercise and maintaining it are not the same behavioral challenge. Insufficient physical activity remains widespread among adolescents worldwide (Guthold et al., 2020). Schools, parents, peers, and organized programs may encourage young people to participate in sport or exercise (Logan et al., 2019; Burke et al., 2024), but such support does not necessarily lead to continued participation. Maintaining regular exercise becomes more difficult when it competes with academic demands, fatigue, social evaluation, setbacks, or declining interest (Thompson et al., 2024; Laursen et al., 2026). Previous studies have consistently identified enjoyment, confidence, social support, and self-determined motivation as important factors in sustained exercise participation among adolescents (Dishman et al., 2005; H. Chen et al., 2017; M. Schneider, 2018). The central issue, therefore, is not only why adolescents begin to exercise, but also how exercise comes to be experienced as sufficiently positive and personally meaningful for them to continue.

Emotional intelligence (EI) offers one perspective for understanding why adolescents respond differently to the emotional demands of exercise. Salovey and Mayer (1990) initially defined EI in terms of monitoring and distinguishing emotions in oneself and others and using emotional information to guide thought and action. Later work further emphasized the perception, understanding, use, and regulation of emotion (Mayer et al., 2016). These processes are relevant to exercise because participation does not always produce positive emotional experiences. Adolescents may encounter physical discomfort, frustration after poor performance, anxiety about evaluation by others, or a gradual decline in motivation (Hortigüela-Alcalá et al., 2021; J. Chen et al., 2024). Their responses to these experiences may be associated with whether exercise remains manageable and rewarding or gradually becomes something they avoid (Lieberman, 2020; Lopez-Garrido, 2023). EI may therefore be relevant to exercise adherence because it shapes how adolescents interpret and manage the emotional experiences that accompany continued participation.

This connection is especially important in sport and exercise settings, where emotional demands are immediate, repeated, and often social in nature. Competition can involve both excitement and pressure. Cooperation requires adolescents to attend to teammates’ reactions as well as their own emotions. Peer comparison may increase confidence in one situation but produce embarrassment or frustration in another (Crone & Fuligni, 2020; Jekauc et al., 2021; White et al., 2021). Success, failure, fatigue, and changes in arousal may also occur within a single exercise session (Butler, 2020; Lorcery et al., 2024). Meyer and Fletcher (2007) therefore argued that EI in sport should be understood in relation to the specific demands of the sporting environment rather than assumed to function identically across contexts. Laborde et al. (2016) likewise reported associations between EI and a range of psychological and behavioral outcomes in sport, while also noting substantial variation in how the construct had been defined and measured. More recently, the Chinese Sports Emotional Intelligence Scale (SEIS) was developed to assess sport-specific emotional functioning across four dimensions: appraisal of others’ emotions, self-emotion management, use of emotion, and social skills (J. Zhang et al., 2022). Within this context, the present study defines sports emotional intelligence as adolescents’ perceived capacity to understand, manage, and use emotions effectively during sport and exercise.

Existing findings provide preliminary support for an association between sports emotional intelligence and exercise-related behavior. In the validation study of the Chinese SEIS, higher scores were associated with greater exercise engagement, commitment, and adherence (J. Zhang et al., 2022). Similar patterns have been reported in other populations. Among athletes, several dimensions of EI have been linked to more autonomous forms of sport motivation (Sukys et al., 2019). An et al. (2024) also identified a chain indirect pathway involving sports emotional intelligence and exercise self-efficacy among university students. More recently, Ruiz-Ariza et al. (2026) found cross-sectional associations between weekly moderate-to-vigorous physical activity and selected dimensions of EI in Spanish adolescents. Together, these studies suggest that emotional functioning may be relevant to sustained participation in exercise.

However, the existing evidence does not establish the direction of this relationship. Regular participation in sport may shape adolescents’ perceptions of their emotional competence, which in turn may influence how they respond to exercise. Reciprocal effects are therefore possible. The present study adopts a more specific proposition. Adolescents who perceive themselves as better able to recognize and regulate exercise-related emotions may be less likely to let a single unpleasant experience determine their broader evaluation of exercise. Fatigue after a demanding session, disappointment after poor performance, or embarrassment in front of peers may then have less influence on whether they continue to participate. Accordingly, H1 is proposed: sports emotional intelligence was expected to be positively associated with exercise adherence.

The expected association between sports emotional intelligence and exercise adherence is only a starting point. A direct relationship does not explain how emotional functioning becomes relevant to continued exercise. One possible pathway is through the affective quality of the exercise experience itself. Research outside the exercise context offers some support for this possibility. Llamas-Díaz et al. (2023), for example, found that emotional intelligence was associated with adolescents’ subjective happiness through positive and negative affect. Although their study used a different measure of emotional intelligence and did not examine exercise behavior, the broader implication is relevant here. Emotional functioning may be associated with later adjustment, partly through emotional experiences that co-occur with it.

In the present study, exercise enjoyment is proposed as the first stage of this process. Enjoyment is more than the immediate pleasure or discomfort experienced during physical exertion (Hutchinson et al., 2020; Mikaelsson et al., 2020). Affective models of physical activity distinguish immediate affective responses from the evaluations that people later remember, anticipate, and attach to an activity (Stevens et al., 2020; Tisza & Markopoulos, 2023). Exercise enjoyment, therefore, reflects a broader judgment that participation is interesting, rewarding, or worth repeating (Rodrigues et al., 2020; Feil et al., 2025). This distinction is important because an exercise session may contain both pleasant and unpleasant moments while still being evaluated positively overall (Bastos et al., 2025; Taylor et al., 2025).

The Affective–Reflective Theory of physical inactivity and exercise provides a useful explanation for this process. The theory proposes that previous exercise experiences leave affective valuations that can influence later tendencies to approach or avoid exercise (Brand & Ekkekakis, 2018; Brand & Cheval, 2019). From this perspective, sports emotional intelligence and exercise enjoyment are related but conceptually distinct. Sports emotional intelligence concerns adolescents’ recognition and management of emotional experiences during exercise (Kopp & Jekauc, 2025; Gakh et al., 2025). Enjoyment reflects how the activity is evaluated after those experiences have been processed (Davidson et al., 2023; Altın et al., 2025). Emotional regulation cannot remove physical exertion, disappointment, or embarrassment. It may, however, prevent a temporary negative experience from dominating the meaning of the entire session. This leaves room for other aspects of participation, such as mastery, social connection, and accomplishment, to contribute to a more positive overall evaluation.

Evidence from adolescent physical activity research supports the importance of such evaluations. In a school-based randomized intervention, changes in enjoyment partly accounted for changes in physical activity among adolescent girls (Dishman et al., 2005). Among Chinese high-school students, exercise enjoyment mediated part of the association between peer support and physical activity (H. Chen et al., 2017). M. L. Schneider and Kwan (2013) also linked affective responses and psychological need satisfaction with intrinsic motivation in adolescents. M. Schneider (2018) later found that intrinsic motivation helped connect exercise-related affect with physical activity. These studies differ in population, design, and measurement, so they do not provide identical tests of the present pathway. Nevertheless, they converge on a common point: exercise experiences that are evaluated as positive or worthwhile are more likely to support continued participation. Accordingly, sports emotional intelligence was expected to show a positive indirect association with exercise adherence through exercise enjoyment (H2).

Exercise maintenance may also depend on something more stable than the evaluation of a single exercise experience: exercise identity. Exercise identity refers to the extent to which being physically active or being a regular exerciser becomes part of an individual’s self-concept (Anderson & Cychosz, 1994). Identity does not determine behavior automatically. However, when adolescents begin to see exercise as part of who they are, participation may take on a more personal meaning (Su et al., 2024; K. Chen et al., 2026). This may be associated with maintaining the personal relevance of exercise even when a particular session is tiring, frustrating, or less enjoyable than expected (Shi et al., 2024; Rhodes et al., 2025). Rhodes et al. (2016) reported a strong association between physical activity identity and physical activity, and identified previous behavior, affective judgments, and autonomous motivation as possible antecedents of identity development. More recently, Porter et al. (2024) used a four-wave cross-lagged design in late adolescence and found that exercise identity prospectively predicted subsequent physical activity. This finding is particularly relevant during adolescence, when patterns of physical activity often become less stable.

Direct evidence linking sports emotional intelligence with exercise identity remains limited. For this reason, the proposed relationship is treated here as a hypothesis rather than an established pathway. Self-determination theory (SDT) provides one explanation for how such a relationship may develop. According to SDT, repeated behavior can become progressively less dependent on external demands and more closely aligned with personal values and the self (Ryan & Deci, 2000; Teixeira et al., 2012). In exercise settings, adolescents frequently encounter frustration, poor performance, social comparison, and other emotionally challenging experiences (Yang & Lu, 2025; Ruiz-Ranz & Asín-Izquierdo, 2025). Those who are better able to understand and regulate these emotions may be less likely to interpret a negative episode as evidence that exercise does not suit them. Over time, this may make it easier to maintain a consistent view of oneself as someone who exercises.

Related findings provide some support for this possibility, although they do not directly test the proposed pathway. Dimensions of EI have been associated with more autonomous sport motivation (Sukys et al., 2019), while exercise identity has also been linked to self-determined motivational processes (Vlachopoulos et al., 2011; Ntoumanis et al., 2018). Importantly, Ntoumanis et al. (2018) found that changes in motivation and exercise identity were related over time rather than following a simple one-directional sequence. The relationship between emotional functioning, motivation, and identity is therefore likely to develop gradually and reciprocally. Nevertheless, within the present model, adolescents with higher sports emotional intelligence were expected to report stronger exercise identity, which in turn would be associated with greater exercise adherence. Exercise identity was therefore expected to mediate the association between sports emotional intelligence and exercise adherence (H3).

Enjoying exercise does not automatically mean that exercise becomes part of an adolescent’s sense of self (Palmer et al., 2020; Su et al., 2024). A more plausible process is gradual. When exercise is experienced positively, adolescents may be more willing to participate again (He et al., 2022). Repeated participation then provides further experience of choosing, performing, and returning to exercise (Kwan et al., 2018; Sutter et al., 2022). Over time, these repeated experiences may contribute to a more stable sense of oneself as a person who exercises.

This sequence is consistent with self-determination theory, which distinguishes between enjoying an activity for its own sake and integrating that activity more fully into one’s values and sense of self. These processes are related, but they are not identical. Previous findings also provide indirect support for this ordering. Ntoumanis et al. (2018) showed that motivation and exercise identity develop in relation to one another over time. Among Chinese adolescents, Dong et al. (2024) linked subjective exercise experience, intrinsic motivation, role identity, and exercise behavior. Porter et al. (2024) further found that exercise identity prospectively predicted later physical activity in late adolescence. These studies do not directly demonstrate that exercise enjoyment leads to exercise identity. They do, however, suggest that positive exercise experiences, motivational processes, and identity are closely connected.

The proposed ordering is therefore theoretically plausible based on existing theories and previous findings. However, because the present study uses a cross-sectional design, this ordering should be interpreted as a theoretically informed statistical model rather than evidence of temporal progression. Alternative explanations and reciprocal associations among sports emotional intelligence, exercise enjoyment, exercise identity, and exercise adherence remain possible. Accordingly, H4 predicted a significant chain indirect association in which sports emotional intelligence was statistically related to exercise adherence through the sequentially specified associations involving exercise enjoyment and exercise identity.

## 2. Materials and Methods

### 2.1. Participants and Procedure

In this study, an online survey system was employed from March to June 2026 in Anhui Province, Henan Province, the Guangxi Zhuang Autonomous Region, and Chongqing Municipality. A school-based convenience sampling approach was adopted. In each school, one class was selected from each grade level (Grades 7–12), resulting in 24 participating classes from four schools. School stage was categorized as junior high school (Grades 7–9) and senior high school (Grades 10–12). Questionnaires were distributed online during class periods with the assistance of teachers. Participation was voluntary and anonymous.

A total of 1462 questionnaires were initially collected. Data screening was conducted before statistical analysis. Responses were excluded if they showed careless response patterns or insufficient completion time. Specifically, 106 questionnaires were removed due to patterned responses (e.g., identical response selections across items), and 78 questionnaires were excluded because they were completed in less than 120 s. After screening, 1278 valid questionnaires were retained for analysis.

The final analytic sample included 1278 adolescents aged 12–18 years (M = 14.37, SD = 1.68), comprising 651 males (50.9%) and 627 females (49.1%). Participants were distributed across Grades 7–12 as follows: 211 (16.5%) in Grade 7, 246 (19.2%) in Grade 8, 232 (18.2%) in Grade 9, 217 (17.0%) in Grade 10, 199 (15.6%) in Grade 11, and 173 (13.5%) in Grade 12. A total of 280 participants (21.9%) reported less than 3 months of exercise experience, 220 (17.2%) reported 3–6 months, 212 (16.6%) reported 6–12 months, 236 (18.5%) reported 1–2 years, and 330 (25.8%) reported more than 2 years.

Removed for double-blind review. All participants agreed to participate voluntarily, with informed consent obtained when they filled in the survey, and they were able to withdraw from the study at any time. The questionnaire was designed and applied to ensure the anonymity of participants. The data were confidential, and participation was anonymous without any potential risk to the integrity of the subjects.

### 2.2. Measures

#### 2.2.1. Sports Emotional Intelligence

Sports emotional intelligence was assessed using the Chinese Sports Emotional Intelligence Scale (SEIS; J. Zhang et al., 2022). The scale contains 14 items across four dimensions: appraisal of others’ emotions (4 items), self-emotion management (4 items), use of emotion (3 items), and social skills (3 items). Responses were recorded on a 7-point Likert scale, with higher scores indicating stronger perceived emotional functioning in sport and exercise. In this study, SEIS scores were interpreted as reflecting adolescents’ perceived sport-specific emotional competence. Cronbach’s α was 0.898.

#### 2.2.2. Exercise Enjoyment

Exercise enjoyment was measured using the seven-item Chinese short form of the Physical Activity Enjoyment Scale (S-PACESC) developed and validated by H. Chen et al. (2019). The scale consists of one dimension with seven items designed to assess the extent to which individuals experience physical activity as enjoyable, interesting, and positively valued. A five-point Likert scale was used, with higher scores indicating greater exercise enjoyment. In the present study, Cronbach’s α for the scale was 0.895.

#### 2.2.3. Exercise Identity

Exercise identity was measured using the nine-item Exercise Identity Scale (EIS) developed by Anderson and Cychosz (1994), following the Chinese adaptation framework reported by Li et al. (2019). The scale consists of one dimension with nine items assessing the extent to which regular exercise is incorporated into an individual’s self-concept and role identity. A seven-point Likert scale was used, with higher scores indicating a stronger exercise identity. In the present study, Cronbach’s α for the scale was 0.913.

#### 2.2.4. Exercise Adherence

Exercise adherence was assessed using the six-item Exercise Adherence Scale described by Liu et al. (2011). The scale consists of one dimension with six items measuring adolescents’ tendency to maintain regular and persistent exercise participation. A five-point Likert scale was used, with higher scores indicating stronger exercise adherence. In the present study, Cronbach’s α for the scale was 0.875.

### 2.3. Statistical Analysis

Statistical analyses were conducted using SPSS 25.0 and Mplus 8.1. First, descriptive statistics, reliability analyses, and Pearson correlation analyses were performed using SPSS 25.0. Confirmatory factor analysis (CFA) and structural equation modeling (SEM) were conducted using Mplus 8.1 with maximum likelihood (ML) estimation. The measurement items were assessed using Likert-type response scales, and item scores were aggregated into composite scores for each construct (sports emotional intelligence, exercise enjoyment, exercise identity, and exercise adherence), which were treated as approximately continuous variables in the SEM analyses. Convergent validity was assessed using composite reliability (CR) and average variance extracted (AVE). Discriminant validity was evaluated using the Fornell–Larcker criterion and the heterotrait–monotrait ratio (HTMT).

Model fit was evaluated using multiple indices, including χ^2^/df, comparative fit index (CFI), Tucker–Lewis index (TLI), root mean square error of approximation (RMSEA), and standardized root mean square residual (SRMR). Bootstrap procedures with 5000 resamples were used to examine indirect associations and generate bias-corrected confidence intervals. Before analysis, questionnaires were screened for invalid responses. Cases with patterned responses (N = 106) and completion times shorter than 120 s (N = 78) were excluded. After data screening, 1278 valid responses were retained. No missing data remained in the final dataset; therefore, missing-data imputation was not required.

## 3. Results

### 3.1. Common-Method Bias Screening

The Harmon one-way method was used to test for common method bias. An exploratory factor analysis of all questionnaire questions revealed that the variance explained by the first factor without rotation was 32.96%, less than the critical criterion of 40%. This finding indicated that no single factor dominated the covariance among the measured variables (L. Zhang et al., 2024). However, because Harman’s single-factor test provides only a limited assessment, it cannot completely exclude the possibility of common method variance. Therefore, the findings should be interpreted with consideration of the self-report nature of the measures.

Given that all variables were collected through self-report questionnaires at a single time point, potential shared method variance may exist. Nevertheless, the use of validated scales, anonymous data collection procedures, and confirmatory factor analysis provided additional support for the reliability and validity of the measurement model.

### 3.2. Measurement Model Assessment

Confirmatory factor analysis (CFA) was conducted to evaluate the construct validity of the measurement model. The hypothesized four-factor model, including sports emotional intelligence, exercise enjoyment, exercise identity, and exercise adherence, demonstrated satisfactory fit to the data (χ^2^/df = 2.45, RMSEA = 0.034, CFI = 0.961, TLI = 0.958, SRMR = 0.029).

All standardized factor loadings were significant and ranged from 0.568 to 0.774. The Cronbach’s alpha coefficients ranged from 0.875 to 0.913, indicating satisfactory internal consistency. Composite reliability values exceeded the recommended criterion of 0.70 (see Table 1). Although the AVE value of sports emotional intelligence was slightly below the conventional threshold of 0.50, the construct demonstrated satisfactory composite reliability and factor loadings, suggesting acceptable convergent validity.

Discriminant validity was examined using the heterotrait–monotrait ratio (HTMT). All HTMT values ranged from 0.442 to 0.684, below the recommended threshold of 0.85 (see Table 2), indicating adequate discriminant validity among the four constructs (Fornell & Larcker, 1981; Henseler et al., 2015).

### 3.3. Descriptive Statistics and Correlations

The results of the descriptive statistics and correlation analysis showed that sports emotional intelligence, exercise enjoyment, exercise identity, and exercise adherence were all significantly and positively correlated with each other (see Table 3).

### 3.4. Chain Mediation Model Test

Structural equation modeling was used to examine the mediating roles of exercise enjoyment and exercise identity in the relationship between sports emotional intelligence and exercise adherence. First, the fit indices of the hypothesized model were assessed, and the results showed that the hypothesized model fit well (CFI = 0.909, TLI = 0.975, RMSEA = 0.006, and SRMR = 0.017).

**Figure 1:**
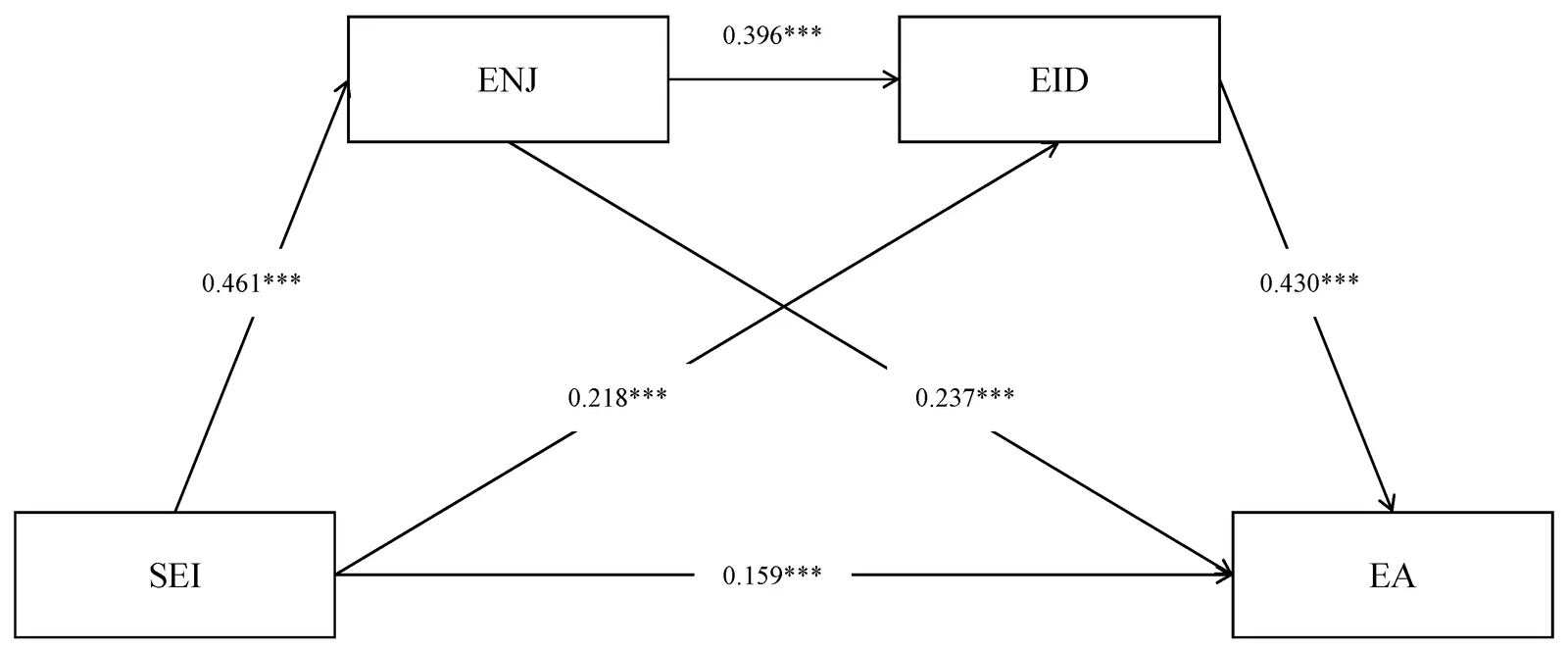
Proposed serial mediation model. Note: *** *p* < 0.001.

Second, the mediating effects were tested. The standardized path estimates are presented in Table 4 and Figure 1. Sports emotional intelligence was significantly and positively associated with exercise enjoyment (β = 0.461, t = 18.55, *p* < 0.001). Sports emotional intelligence and exercise enjoyment were both positively associated with exercise identity (β = 0.218, t = 8.18, *p* < 0.001; β = 0.396, t = 14.82, *p* < 0.001, respectively). When exercise enjoyment and exercise identity were entered simultaneously, sports emotional intelligence remained positively associated with exercise adherence (β = 0.159, t = 6.64, *p* < 0.001). Exercise enjoyment (β = 0.237, t = 9.41, *p* < 0.001) and exercise identity (β = 0.430, t = 17.64, *p* < 0.001) were also significant positive predictors of exercise adherence. In addition, the total association between sports emotional intelligence and exercise adherence was significant (β = 0.440, t = 17.52, *p* < 0.001), providing support for H1.

A bias-corrected Bootstrap procedure with 5000 resamples was used to examine the indirect effects. As shown in Table 5, the 95% confidence interval for the indirect association through exercise enjoyment did not include 0 (β = 0.109). Thus, exercise enjoyment significantly mediated the association between sports emotional intelligence and exercise adherence, providing statistical support for H2. The indirect association through exercise identity was also significant (β = 0.094), providing statistical support for H3. More importantly, the statistically specified chain indirect association from sports emotional intelligence to exercise adherence involving exercise enjoyment and exercise identity was significant (β = 0.079). The confidence interval did not include 0, providing statistical support for H4.

The total indirect effect of the three pathways was 0.282. After exercise enjoyment and exercise identity were included in the model, the direct association between sports emotional intelligence and exercise adherence remained significant (β = 0.159). The results therefore indicate partial statistical mediation through exercise enjoyment and exercise identity.

## 4. Discussion

This study examined the association between sports emotional intelligence and exercise adherence among adolescents, with particular attention to the mediating roles of exercise enjoyment and exercise identity. The results supported all four hypotheses. Sports emotional intelligence was positively associated with exercise adherence, supporting H1. Exercise enjoyment and exercise identity each showed significant indirect associations between sports emotional intelligence and adherence, supporting H2 and H3. The chain indirect pathway from sports emotional intelligence to exercise enjoyment, then to exercise identity and exercise adherence, was also significant, supporting H4.

These findings suggest that the relationship between sports emotional intelligence and exercise adherence is not limited to a direct association. Much of this relationship was shared with how adolescents evaluate exercise and the extent to which exercise becomes part of their self-concept. The total standardized association between sports emotional intelligence and adherence was 0.440, whereas the direct coefficient decreased to 0.159 after exercise enjoyment and exercise identity were included. The three indirect pathways together accounted for a substantial part of the overall association. At the same time, the remaining direct association indicates that enjoyment and identity do not capture all of the processes through which sports emotional intelligence may relate to continued exercise.

### 4.1. The Association Between Sports Emotional Intelligence and Exercise Adherence

Sports emotional intelligence was positively associated with exercise adherence, which is consistent with previous studies linking emotional functioning in sport with sustained participation. J. Zhang et al. (2022), for example, reported associations between sports emotional intelligence and exercise engagement, commitment, and adherence. An et al. (2024) also placed sports emotional intelligence within an indirect pathway leading to exercise adherence among university students. In addition, dimensions of emotional intelligence have been associated with more autonomous forms of sport motivation (Sukys et al., 2019).

One explanation for the present finding is that exercise frequently produces emotional experiences that can either support or disrupt continued participation. Adolescents may experience disappointment after poor performance, embarrassment when comparing themselves with peers, frustration when progress is slower than expected, or discomfort during demanding exercise (Guo et al., 2025; K. Chen et al., 2026). These experiences do not necessarily correspond to withdrawal. Their influence may depend partly on how they are understood and managed (Sette et al., 2023). Adolescents who perceive themselves as better able to recognize and regulate exercise-related emotions may be less likely to interpret one difficult experience as evidence that exercise is unpleasant or unsuitable for them.

The reduction in the sports emotional intelligence coefficient from 0.440 to 0.159 after the two mediators were included is therefore informative. It suggests that a considerable part of its association with adherence is linked to exercise enjoyment and exercise identity. However, the remaining direct association was still significant. Other psychological processes may therefore also be involved. For example, emotional competence may contribute to persistence through coping with competitive pressure, managing arousal, maintaining interpersonal relationships in sport, or strengthening confidence in dealing with exercise challenges (An et al., 2024; Guillaume et al., 2025; Alexander, 2025). Continued exercise may itself provide repeated opportunities for adolescents to learn how to respond to frustration, social evaluation, and physical discomfort. Regular participation could therefore strengthen perceived emotional competence over time.

### 4.2. Separate Mediating Roles of Exercise Enjoyment and Exercise Identity

The results showed that exercise enjoyment and exercise identity each carried a significant indirect association between sports emotional intelligence and exercise adherence. These two pathways indicate that affective evaluation and self-concept may represent different psychological processes linking emotional functioning with continued exercise.

Sports emotional intelligence was positively associated with enjoyment, and enjoyment was in turn positively associated with adherence. This finding is consistent with the idea that emotional regulation may influence not whether exercise is physically demanding, but how the overall experience is evaluated. Exercise may still involve fatigue, frustration, or temporary embarrassment. Adolescents who manage these experiences effectively may be less likely to allow a negative moment to dominate their overall evaluation of the activity (Zhou et al., 2023; G. Zhang et al., 2024). Positive aspects of participation, such as mastery, social interaction, achievement, or satisfaction, may therefore remain more salient. This interpretation is compatible with the Affective–Reflective Theory of physical inactivity and exercise, which proposes that previous exercise experiences acquire affective value and influence later tendencies to approach or avoid exercise (Brand & Ekkekakis, 2018; Brand & Cheval, 2019). Previous empirical findings also support the importance of enjoyment for continued activity. Dishman et al. (2005) found that changes in enjoyment partly accounted for changes in physical activity among adolescent girls. H. Chen et al. (2017) similarly reported that enjoyment mediated part of the association between peer support and physical activity among Chinese adolescents. These findings suggest that exercise experiences perceived as enjoyable or rewarding are more likely to encourage future participation.

Exercise identity provided a second indirect pathway, with an indirect effect of 0.094. In the final model, exercise identity also showed the strongest association with adherence (β = 0.430). This result is consistent with the idea that exercise identity represents a relatively stable connection between exercise and the self. When adolescents begin to regard regular exercise as part of who they are, participation may retain personal importance even when a particular session is difficult or less enjoyable than expected (González et al., 2019; Ruiz-Ranz & Asín-Izquierdo, 2025).

Previous research supports the relevance of identity to sustained physical activity. Rhodes et al. (2016) reported a robust association between physical activity identity and activity behavior. More importantly, Porter et al. (2024) found prospective evidence that exercise identity predicted later physical activity during late adolescence. From the perspective of self-determination theory, repeated participation can also become increasingly self-endorsed and integrated with personal values and the self (Ryan & Deci, 2000; Teixeira et al., 2012). Sports emotional intelligence may be associated with adolescents’ ways of dealing with negative exercise experiences without allowing those experiences to undermine their broader exercise-related self-view. The findings therefore suggest different roles for enjoyment and identity. Enjoyment concerns how positively exercise is experienced and evaluated, whereas identity concerns whether exercising has become part of the individual’s self-definition.

### 4.3. Chain Mediating Role of Exercise Enjoyment and Exercise Identity

A further finding was the significant chain indirect pathway from sports emotional intelligence to exercise adherence through exercise enjoyment and then exercise identity. The standardized indirect effect was 0.079, and its 95% bootstrap confidence interval did not include 0. A possible interpretation is that adolescents who perceive themselves as capable of managing exercise-related emotions are more likely to preserve a positive overall evaluation of exercise, even when participation includes discomfort or setbacks. Positive exercise experiences may then make voluntary re-engagement more likely (Ralf et al., 2025; Stephen et al., 2025). Repeated participation provides further opportunities for exercise to become personally meaningful and increasingly integrated into the self (Davis et al., 2021; Y. Zhang et al., 2025). Once adolescents begin to see themselves as people who exercise, continued participation may become more consistent with that self-definition and therefore easier to maintain (Shao & Zhou, 2023; Morgan et al., 2024).

Self-determination theory provides a useful framework for understanding this sequence. Enjoying an activity and incorporating it into one’s values and identity are related processes, but they are not equivalent. An adolescent may enjoy a particular activity without yet defining exercise as part of who they are. With repeated and self-endorsed participation, however, exercise may gradually acquire greater personal significance. Previous studies linking self-determined motivation with exercise identity are consistent with this possibility (Vlachopoulos et al., 2011; Ntoumanis et al., 2018). Ntoumanis et al. (2018) also showed that motivation and exercise identity change together over time, while Dong et al. (2024) reported relationships among subjective exercise experience, intrinsic motivation, role identity, and exercise behavior in Chinese adolescents.

The chain pathway therefore adds to the separate mediation findings by suggesting that affective evaluation and identity may be connected rather than operating independently. Sports emotional intelligence may be associated with whether exercise experiences are evaluated positively; these evaluations may, over repeated participation, contribute to how adolescents define themselves in relation to exercise. Exercise identity may then provide a relatively stable basis for continued participation.

Overall, the present study suggests that the chain mediation model provides a more detailed account of how sports emotional intelligence is associated with exercise adherence among adolescents. Sports emotional intelligence was related to adherence both directly and indirectly through exercise enjoyment and exercise identity. Adolescents with higher sports emotional intelligence may be better able to manage negative emotional experiences during exercise and maintain a more positive evaluation of participation. Greater enjoyment may, in turn, encourage repeated and self-endorsed engagement, providing the basis for exercise to become more strongly incorporated into the self-concept. As exercise identity becomes stronger, adolescents may be more likely to maintain regular participation over time. These findings therefore support a sequential pathway in which sports emotional intelligence is associated with exercise adherence through exercise enjoyment and subsequent exercise identity, while also indicating that additional psychological statistical mechanisms may contribute to the remaining direct association.

Although the proposed serial mediation model was supported statistically, the findings should not be interpreted as demonstrating a temporal developmental sequence. Because all variables were measured at a single time point, the directionality among sports emotional intelligence, exercise enjoyment, exercise identity, and exercise adherence cannot be determined. Alternative explanations are possible. For example, adolescents who regularly engage in exercise may gradually develop stronger exercise identity and more positive emotional experiences, while repeated participation may also provide opportunities to enhance perceived emotional competence. Therefore, longitudinal and experimental studies are needed to further examine the dynamic and reciprocal relationships among these constructs.

### 4.4. Theoretical and Practical Implications

The findings may provide several potential implications for physical education practice, although they should be interpreted cautiously given the cross-sectional design. The observed associations suggest that sports emotional intelligence, exercise enjoyment, and exercise identity may represent important psychological factors related to adolescents’ exercise adherence.

For teachers and coaches, creating supportive exercise environments that encourage positive emotional experiences and personal engagement may be a promising direction. For example, activities designed to help adolescents recognize and regulate exercise-related emotions, experience competence, and develop a stronger connection with physical activity could be explored in future intervention studies. However, whether such approaches can directly improve exercise adherence requires further empirical examination.

In addition, interventions that focus only on increasing exercise participation may not be sufficient. Future programs may consider incorporating psychological components, such as enjoyment enhancement and identity development, while evaluating their effectiveness through longitudinal or experimental designs.

### 4.5. Limitations and Future Directions

Several limitations should be considered. First, the cross-sectional design prevents the determination of temporal order and causal relationships among sports emotional intelligence, exercise enjoyment, exercise identity, and exercise adherence. Although the proposed serial mediation model was theoretically supported, the findings should be interpreted as associations rather than causal effects. Future longitudinal studies are needed to further examine the dynamic relationships among these variables.

Second, because all measures were collected through self-report questionnaires at a single time point, common method bias and response bias cannot be completely excluded. Future research could incorporate objective behavioral measures and multiple sources of information to improve the robustness of findings.

Third, alternative explanations and unmeasured factors should also be considered. For example, regular exercise participation may itself contribute to the development of exercise identity, positive exercise experiences, and emotional competence. Other factors, such as social support, motivation, and perceived competence, may also influence adolescents’ exercise adherence. Moreover, the sample was limited to Chinese adolescents, which may restrict the generalizability of the findings to other populations and cultural contexts. Future studies involving diverse samples and longitudinal designs are encouraged.

Finally, because participants were recruited through classes within schools, the data may contain potential clustering effects. Although the present study focused on individual-level associations, future research should consider multilevel modeling or cluster-robust estimation approaches to account for nested educational structures.

## 5. Conclusions

This study examined the associations among sports emotional intelligence, exercise enjoyment, exercise identity, and exercise adherence in adolescents. The findings indicated that sports emotional intelligence was positively associated with exercise adherence. Furthermore, exercise enjoyment and exercise identity showed significant indirect associations in the relationship between sports emotional intelligence and exercise adherence. These findings suggest that emotional and identity-related factors may be important correlates of adolescents’ exercise adherence within the proposed theoretical model.

## Figures and Tables

**Table 1:** Reliability and convergent validity.

Construct	CR	AVE
SEI	0.898	0.387
ENJ	0.895	0.550
EID	0.913	0.540
EA	0.875	0.539

Note: SEI = sports emotional intelligence; ENJ = exercise enjoyment; EID = exercise identity; EA = exercise adherence.

**Table 2:** HTMT discriminant validity.

	SEI	ENJ	EID	EA
SEI	—	0.514	0.442	0.497
ENJ		—	0.549	0.591
EID			—	0.684
EA				—

**Table 3:** Results of descriptive statistics and correlation analysis of each variable.

Variable	M	SD	SEI	ENJ	EID	EA
SEI	5.08	1.06	1			
ENJ	3.46	0.97	0.461 ***	1		
EID	4.84	1.27	0.401 ***	0.496 ***	1	
EA	3.30	0.98	0.440 ***	0.524 ***	0.612 ***	1

Note: N = 1278; *** *p* < 0.001.

**Table 4:** Standardized regression paths in the chain mediation model.

Outcome	Predictor	R^2^	β	SE	t
ENJ	SEI	0.212	0.461	0.025	18.55 ***
EID	SEI	0.284	0.218	0.027	8.18 ***
	ENJ		0.396	0.027	14.82 ***
EA	SEI	0.457	0.159	0.024	6.64 ***
	ENJ		0.237	0.025	9.41 ***
	EID		0.430	0.024	17.64 ***
EA (total effect)	SEI	0.194	0.440	0.025	17.52 ***

Note: N = 1278. Standardized regression coefficients are reported. *** *p* < 0.001.

**Table 5:** Bootstrap indirect effects.

Path	Point Estimate	Bootstrap SE	Bias-Corrected 95% CI	Hypothesis
SEI → ENJ → EA	0.109	0.013	0.085, 0.136	H2 supported
SEI → EID → EA	0.094	0.013	0.070, 0.120	H3 supported
SEI → ENJ → EID → EA	0.079	0.008	0.064, 0.095	H4 supported
Total indirect effect	0.282	0.017	0.249, 0.315	

Note: The sequential paths represent statistically specified indirect associations and should not be interpreted as evidence of temporal causality.

## Data Availability

The data presented in this study are available on request from the corresponding author.

## References

[B1-jintelligence-14-00222] Alexander T. L. (2025). Integrative self-regulation model for sport and exercise: Theory and implications for comprehensive training. Performance Enhancement & Health.

[B2-jintelligence-14-00222] Altın Y., Özkurt B., Küçükibiş H. F. (2025). Examination of the psychometric properties of physical activity enjoyment scale (PACES) based on classical test theory and item response theory. Acta Psychologica.

[B3-jintelligence-14-00222] An D., Pan J., Ran F., Bai D., Zhang J. (2024). Effects of physical exercise input on the exercise adherence of college students: The chain mediating role of sports emotional intelligence and exercise self-efficacy. Journal of Intelligence.

[B4-jintelligence-14-00222] Anderson D. F., Cychosz C. M. (1994). Development of an exercise identity scale. Perceptual and Motor Skills.

[B5-jintelligence-14-00222] Bastos V., Ekkekakis P., Andrade A. J., Teixeira D. S. (2025). The peak and end rule, affect-related cognitions, enjoyment, and exercise frequency: A randomized controlled trial ancillary study. Psychology of Sport and Exercise.

[B6-jintelligence-14-00222] Brand R., Cheval B. (2019). Theories to explain exercise motivation and physical inactivity: Ways of expanding our current theoretical perspective. Frontiers in Psychology.

[B7-jintelligence-14-00222] Brand R., Ekkekakis P. (2018). Affective-reflective theory of physical inactivity and exercise: Foundations and preliminary evidence. German Journal of Exercise and Sport Research.

[B8-jintelligence-14-00222] Burke S., Sharp L. A., Woods D., Paradis K. F. (2024). Enhancing parental support through parent-education programs in youth sport: A systematic review. International Review of Sport and Exercise Psychology.

[B9-jintelligence-14-00222] Butler R. (2020). Sports psychology in action.

[B10-jintelligence-14-00222] Chen H., Dai J., Sun H. (2019). Validation of a Chinese version of the physical activity enjoyment scale: Factorial validity, measurement equivalence, and predictive validity. International Journal of Sport and Exercise Psychology.

[B11-jintelligence-14-00222] Chen H., Sun H., Dai J. (2017). Peer support and adolescents’ physical activity: The mediating roles of self-efficacy and enjoyment. Journal of Pediatric Psychology.

[B12-jintelligence-14-00222] Chen J., Bai Y., Ni W. (2024). Reasons and promotion strategies of physical activity constraints in obese/overweight children and adolescents. Sports Medicine and Health Science.

[B13-jintelligence-14-00222] Chen K., Chen C., Yang J., Li Z. (2026). The impact of peer effects on adolescents’ physical exercise: A meta-synthesis study. Frontiers in Psychology.

[B14-jintelligence-14-00222] Crone E. A., Fuligni A. J. (2020). Self and others in adolescence. Annual Review of Psychology.

[B15-jintelligence-14-00222] Davidson S. S., Keebler J. R., Zhang T., Chaparro B., Szalma J., Frederick C. M. (2023). The development and validation of a universal enjoyment measure: The enjoy scale. Current Psychology.

[B16-jintelligence-14-00222] Davis A. J., MacCarron P., Cohen E. (2021). Social reward and support effects on exercise experiences and performance: Evidence from parkrun. PLoS ONE.

[B17-jintelligence-14-00222] Dishman R. K., Motl R. W., Saunders R., Felton G., Ward D. S., Dowda M., Pate R. R. (2005). Enjoyment mediates effects of a school-based physical-activity intervention. Medicine & Science in Sports & Exercise.

[B18-jintelligence-14-00222] Dong B., Li H., Liu R. (2024). Relationship of role identity, intrinsic motivation, subjective exercise experience, and exercise behavior in adolescents. Journal of Tianjin University of Sport.

[B19-jintelligence-14-00222] Feil K., Fritsch J., Weyland S., Jekauc D. (2025). Examining the role of anticipated enjoyment and intention in predicting attendance in exercise classes. BMC Psychology.

[B20-jintelligence-14-00222] Fornell C., Larcker D. F. (1981). Evaluating structural equation models with unobservable variables and measurement error. Journal of Marketing Research.

[B21-jintelligence-14-00222] Gakh R., Tsykvas R., Maliar E., Korniienko S., Khomulenko B. (2025). Neuroscientific perspectives on emotional intelligence and self-regulation in individual sports. BRAIN. Broad Research in Artificial Intelligence and Neuroscience.

[B22-jintelligence-14-00222] González H. J., Gómez L. M., Pérez T. J. A., Muñoz V. A. J., Andreu C. E. (2019). Perfectly active teenagers. When does physical exercise help psychological well-being in adolescents?. International Journal of Environmental Research and Public Health.

[B23-jintelligence-14-00222] Guillaume L., Guillaume M., Yohan S., Michel N. (2025). Longitudinal relationships between emotional processes and perceived performances among athletes: The moderating effect of emotional intelligence. Performance Enhancement & Health.

[B24-jintelligence-14-00222] Guo C., Chen F., Wang Y. (2025). The psychological impact of competitive sports participation on adolescent athletes: An analysis of coping mechanisms and performance outcomes. Journal of Rational-Emotive & Cognitive-Behavior Therapy.

[B25-jintelligence-14-00222] Guthold R., Stevens G. A., Riley L. M., Bull F. C. (2020). Global trends in insufficient physical activity among adolescents: A pooled analysis of 298 population-based surveys with 1.6 million participants. The Lancet Child & Adolescent Health.

[B26-jintelligence-14-00222] He L., Li Y., Chen Z. (2022). The effect of subjective exercise experience on exercise behavior and amount of exercise in children and adolescents: The mediating effect of exercise commitment. International Journal of Environmental Research and Public Health.

[B27-jintelligence-14-00222] Henseler J., Ringle C. M., Sarstedt M. (2015). A new criterion for assessing discriminant validity in variance-based structural equation modeling. Journal of the Academy of Marketing Science.

[B28-jintelligence-14-00222] Hortigüela-Alcalá D., Barba-Martín R. A., González-Calvo G., Hernando-Garijo A. (2021). ‘I Hate Physical Education’; an analysis of girls’ experiences throughout their school life. Journal of Gender Studies.

[B29-jintelligence-14-00222] Hutchinson J. C., Zenko Z., Santich S., Dalton P. C. (2020). Increasing the pleasure and enjoyment of exercise: A novel resistance-training protocol. Journal of Sport and Exercise Psychology.

[B30-jintelligence-14-00222] Jekauc D., Fritsch J., Latinjak A. T. (2021). Toward a theory of emotions in competitive sports. Frontiers in Psychology.

[B31-jintelligence-14-00222] Kopp A., Jekauc D. (2025). Trait emotional intelligence in competitive sports: Are there differences in dimensions of emotional intelligence when comparing different sports?. BMC Psychology.

[B32-jintelligence-14-00222] Kwan B. M., Bryan A. D., Sheeran P. (2018). The dynamics of success and failure: How post-behaviour evaluations relate to subsequent exercise intentions and behaviour. Psychology & Health.

[B33-jintelligence-14-00222] Laborde S., Dosseville F., Allen M. S. (2016). Emotional intelligence in sport and exercise: A systematic review. Scandinavian Journal of Medicine & Science in Sports.

[B34-jintelligence-14-00222] Laursen C. B., Bang C. W., Eggertsen C. N., Hagstrøm S., Haslund-Thomsen H. L., Agergaard S. (2026). Embodied well-being: Energy, exhaustion, and bodily difference among danish school children. Social Science & Medicine.

[B35-jintelligence-14-00222] Li M., Yang J., Ren Y. (2019). Reliability and validity of the Chinese version of the exercise identity scale in a college student sample. Chinese Journal of Clinical Psychology.

[B36-jintelligence-14-00222] Lieberman D. (2020). Exercised: Why something we never evolved to do is healthy and rewarding.

[B37-jintelligence-14-00222] Liu W., Zhou C., Sun J. (2011). Effects of adolescents’ outdoor exercise motivation on exercise adherence: The mediating role of exercise climate. China Sport Science.

[B38-jintelligence-14-00222] Llamas-Díaz D., Cabello R., Gómez-Leal R., Gutiérrez-Cobo M. J., Megías-Robles A., Fernández-Berrocal P. (2023). Ability emotional intelligence and subjective happiness in adolescents: The role of positive and negative affect. Journal of Intelligence.

[B39-jintelligence-14-00222] Logan K., Cuff S., LaBella C. R., Brooks M. A., Canty G., Diamond A. B., Hennrikus W., Moffatt K., Nemeth B. A., Pengel K. B., Peterson A. R., Stricker P. R., COUNCIL ON SPORTS MEDICINE AND FITNESS (2019). Organized sports for children, preadolescents, and adolescents. Pediatrics.

[B40-jintelligence-14-00222] Lopez-Garrido G. (2023). Bandura’s self-efficacy theory of motivation in psychology. Simply Psychology.

[B41-jintelligence-14-00222] Lorcery A., André N., Benraïss A., Pingault M., Mirabelli F., Audiffren M. (2024). Engagement of mental effort in response to mental fatigue: A psychophysiological analysis. Psychology of Sport and Exercise.

[B42-jintelligence-14-00222] Mayer J. D., Caruso D. R., Salovey P. (2016). The ability model of emotional intelligence: Principles and updates. Emotion Review.

[B43-jintelligence-14-00222] Meyer B. B., Fletcher T. B. (2007). Emotional intelligence: A theoretical overview and implications for research and professional practice in sport psychology. Journal of Applied Sport Psychology.

[B44-jintelligence-14-00222] Mikaelsson K., Rutberg S., Lindqvist A. K., Michaelson P. (2020). Physically inactive adolescents’ experiences of engaging in physical activity. European Journal of Physiotherapy.

[B45-jintelligence-14-00222] Morgan J. A., Bednarz J. M., Semo R., Clark S. R., Schubert K. O. (2024). Long-term recreational exercise patterns in adolescents and young adults: Trajectory predictors and associations with health, mental-health, and educational outcomes. PLoS ONE.

[B46-jintelligence-14-00222] Ntoumanis N., Stenling A., Thøgersen-Ntoumani C., Vlachopoulos S., Lindwall M., Gucciardi D. F., Tsakonitis C. (2018). Longitudinal associations between exercise identity and exercise motivation: A multilevel growth curve model approach. Scandinavian Journal of Medicine & Science in Sports.

[B47-jintelligence-14-00222] Palmer K., Robbins L. B., Ling J. Y., Kao T. S. A., Voskuil V. R., Smith A. L. (2020). Adolescent autonomous motivation for physical activity: A concept analysis. Journal of Pediatric Nursing.

[B48-jintelligence-14-00222] Porter C. D., Kwan M. Y. W., Meca A., Brown D. M. Y. (2024). Exercise identity and physical activity behavior during late adolescence: A four-wave cross-lagged panel model. Psychology of Sport and Exercise.

[B49-jintelligence-14-00222] Ralf B., Gorden S., Panteleimon E. (2025). Affective experiences from exercise. Youth-adult differences and prediction of exercise behavior. Psychology of Sport and Exercise.

[B50-jintelligence-14-00222] Rhodes R. E., Kaushal N., Quinlan A. (2016). Is physical activity a part of who I am? A review and meta-analysis of identity, schema and physical activity. Health Psychology Review.

[B51-jintelligence-14-00222] Rhodes R. E., Wierts C. M., Kullman S., Magel E., Strachan S. (2025). Intervention effects on physical activity identity: A systematic review and meta-analysis. Health Psychology Review.

[B52-jintelligence-14-00222] Rodrigues F., Teixeira D. S., Neiva H. P., Cid L., Monteiro D. (2020). The bright and dark sides of motivation as predictors of enjoyment, intention, and exercise persistence. Scandinavian Journal of Medicine & Science in Sports.

[B53-jintelligence-14-00222] Ruiz-Ariza A., Moral-García J. E., Rusillo-Magdaleno A., Solas-Martínez J. L. (2026). Association between weekly moderate-to-vigorous physical activity and emotional intelligence factors in Spanish adolescents: Perspectives for digital and gamified interventions. Journal of Intelligence.

[B54-jintelligence-14-00222] Ruiz-Ranz E., Asín-Izquierdo I. (2025). Physical activity, exercise, and mental health of healthy adolescents: A review of the last 5 years. Sports Medicine and Health Science.

[B55-jintelligence-14-00222] Ryan R. M., Deci E. L. (2000). Self-determination theory and the facilitation of intrinsic motivation, social development, and well-being. American Psychologist.

[B56-jintelligence-14-00222] Salovey P., Mayer J. D. (1990). Emotional intelligence. Imagination, Cognition and Personality.

[B57-jintelligence-14-00222] Schneider M. (2018). Intrinsic Motivation Mediates the Association between Exercise-Associated Affect and Physical Activity among Adolescents. Frontiers in Psychology.

[B58-jintelligence-14-00222] Schneider M. L., Kwan B. M. (2013). Psychological need satisfaction, intrinsic motivation and affective response to exercise in adolescents. Psychology of Sport and Exercise.

[B59-jintelligence-14-00222] Sette S., Pecora G., Laghi F., Coplan R. J. (2023). Motivations for social withdrawal, mental health, and well-being in emerging adulthood: A person-oriented approach. Behavioral Sciences.

[B60-jintelligence-14-00222] Shao T., Zhou X. (2023). Correlates of physical activity habits in adolescents: A systematic review. Frontiers in Physiology.

[B61-jintelligence-14-00222] Shi L., Jiang L., Zhou S., Zhou W., Yang H. (2024). Self-appreciation is not enough: Exercise identity mediates body appreciation and physical activity and the role of perceived stress. Frontiers in Psychology.

[B62-jintelligence-14-00222] Stephen S., Noel E. B., Gavin B. (2025). Core affective responses to light, moderate, and high intensity resistance exercise: A quasi-experimental study. International Journal of Sport and Exercise Psychology.

[B63-jintelligence-14-00222] Stevens C. J., Baldwin A. S., Bryan A. D., Conner M., Rhodes R. E., Williams D. M. (2020). Affective determinants of physical activity: A conceptual framework and narrative review. Frontiers in Psychology.

[B64-jintelligence-14-00222] Su Z., Zhang Z., Zhou Y. (2024). Impact of exercise atmosphere on adolescents’ exercise behavior: Chain mediating effect of exercise identity and exercise habit. International Journal of Mental Health Promotion.

[B65-jintelligence-14-00222] Sukys S., Tilindienė I., Cesnaitiene V. J., Kreivytė R. (2019). Does emotional intelligence predict athletes’ motivation to participate in sports?. Perceptual and Motor Skills.

[B66-jintelligence-14-00222] Sutter K., Oostwoud W. L., Van B. R. J., Medendorp W. P. (2022). Even well-practiced movements benefit from repetition. Journal of Neurophysiology.

[B67-jintelligence-14-00222] Taylor S., Martin J., Wilson O. W., Elliot L., Bopp M. (2025). The impact of physical activity enjoyment, exercise self-efficacy, recording physical activity, and exercise goal setting on physical activity levels of college students. Recreational Sports Journal.

[B68-jintelligence-14-00222] Teixeira P. J., Carraça E. V., Markland D., Silva M. N., Ryan R. M. (2012). Exercise, physical activity, and self-determination theory: A systematic review. International Journal of Behavioral Nutrition and Physical Activity.

[B69-jintelligence-14-00222] Thompson F., Rongen F., Cowburn I., Till K. (2024). A longitudinal mixed methods case study investigation of the academic, athletic, psychosocial and psychological impacts of being a sport school student athlete: F. Thompson et al. Sports Medicine.

[B70-jintelligence-14-00222] Tisza G., Markopoulos P. (2023). FunQ: Measuring the fun experience of a learning activity with adolescents. Current Psychology.

[B71-jintelligence-14-00222] Vlachopoulos S. P., Kaperoni M., Moustaka F. C. (2011). The relationship of self-determination theory variables to exercise identity. Psychology of Sport and Exercise.

[B72-jintelligence-14-00222] White R. L., Bennie A., Vasconcellos D., Cinelli R., Hilland T., Owen K. B., Lonsdale C. (2021). Self-determination theory in physical education: A systematic review of qualitative studies. Teaching and Teacher Education.

[B73-jintelligence-14-00222] Yang X., Lu L. H. (2025). Effects of physical exercise on negative emotions in adolescents: Sequential mediation through psychological benefits and social self-efficacy. Preventive Medicine Reports.

[B74-jintelligence-14-00222] Zhang G., Feng W., Zhao L., Zhao X., Li T. (2024). The association between physical activity, self-efficacy, stress self-management and mental health among adolescents. Scientific Reports.

[B75-jintelligence-14-00222] Zhang J., Bai D., Qin L., Song P. (2022). The development of the Chinese version of the sports emotional intelligence scale. Frontiers in Psychology.

[B76-jintelligence-14-00222] Zhang L., Bai D. H., Song P. W., Zhang J. (2024). Effects of physical health beliefs on college students’ physical exercise behavior intention: Mediating effects of exercise imagery. BMC Psychology.

[B77-jintelligence-14-00222] Zhang Y., Mu F., Han S., Lou H., Li B., Li R. (2025). Transforming physical exertion into emotional agility via sequential empowerment of resilience and self-belief. Scientific Reports.

[B78-jintelligence-14-00222] Zhou X., Bambling M., Bai X., Edirippulige S. (2023). Chinese school adolescents’ stress experience and coping strategies: A qualitative study. BMC Psychology.

